# Thermally Oxidized Memristor and 1T1R Integration for Selector Function and Low‐Power Memory

**DOI:** 10.1002/advs.202401915

**Published:** 2024-07-03

**Authors:** Zhidong Pan, Jielian Zhang, Xueting Liu, Lei Zhao, Jingyi Ma, Chunlai Luo, Yiming Sun, Zhiying Dan, Wei Gao, Xubing Lu, Jingbo Li, Nengjie Huo

**Affiliations:** ^1^ School of Semiconductor Science and Technology South China Normal University Foshan 528225 P. R. China; ^2^ School of South China Academy of Advanced Optoelectronics South China Normal University Guangzhou 510006 P. R. China; ^3^ College of Optical Science and Engineering Zhejiang University Hangzhou 310027 P. R. China; ^4^ Guangdong Provincial Key Laboratory of Chip and Integration Technology Guangzhou 510631 P. R. China

**Keywords:** 1T1R integration, memristor, resistive switching, thermal oxidation, transistor

## Abstract

Resistive switching memories have garnered significant attention due to their high‐density integration and rapid in‐memory computing beyond von Neumann's architecture. However, significant challenges are posed in practical applications with respect to their manufacturing process complexity, a leakage current of high resistance state (HRS), and the sneak‐path current problem that limits their scalability. Here, a mild‐temperature thermal oxidation technique for the fabrication of low‐power and ultra‐steep memristor based on Ag/TiO_x_/SnO_x_/SnSe_2_/Au architecture is developed. Benefiting from a self‐assembled oxidation layer and the formation/rupture of oxygen vacancy conductive filaments, the device exhibits an exceptional threshold switching behavior with high switch ratio exceeding 10^6^, low threshold voltage of ≈1 V, long‐term retention of >10^4^ s, an ultra‐small subthreshold swing of 2.5 mV decade^−1^ and high air‐stability surpassing 4 months. By decreasing temperature, the device undergoes a transition from unipolar volatile to bipolar nonvolatile characteristics, elucidating the role of oxygen vacancies migration on the resistive switching process. Further, the 1T1R structure is established between a memristor and a 2H‐MoTe_2_ transistor by the van der Waals (vdW) stacking approach, achieving the functionality of selector and multi‐value memory with lower power consumption. This work provides a mild‐thermal oxidation technology for the low‐cost production of high‐performance memristors toward future in‐memory computing applications.

## Graphical abstract

This work develops a mild‐temperature thermal oxidation technique for the fabrication of low‐power and ultra‐steep memristors, exhibiting an exceptional threshold switching behavior due to a self‐assembled oxidation layer and atomic‐level control over the formation/rupture of oxygen vacancy conductive filaments. The novel concept of 1T1R is further proposed by the van der Waals stacking, achieving the functionality of selector and multi‐value memory.

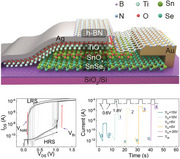

## 1 Introduction

Memristor has been considered the fourth fundamental circuit element in basic circuits due to its inherent advantages of high‐speed switching, low power consumption, and versatile capability of in‐memory computing, showing a great application potential in next‐generation high‐density memory and energy‐efficient neuromorphic computing.^[^ ^1^, ^2^, ^3^, ^4^, ^5^, ^6^, ^7^, ^8^ ^]^ It is widely demonstrated that the resistive switching (RS) behavior of a memristor is determined by the formation and disruption of the internal conductive filaments consisting of metal cations or vacancy defects under the influence of an applied electric field.^[^ ^9^, ^10^, ^11^, ^12^, ^13^ ^]^ Among these, the oxygen vacancy conducting filament‐based memristors have played an important role in the domains of memory, logic devices, ultra‐steep slope transistors neural synapses, etc.^[^ ^14^, ^15^, ^16^, ^17^, ^18^, ^19^ ^]^

In the past several years, oxygen vacancy‐based memristors have been continuously optimized in the aspect of the fabrication process, power consumption, and reliability.^[^ ^20^, ^21^, ^22^, ^23^, ^24^ ^]^ The oxide thin films such as TiO_2_ and HfO_2_, serving as the essential components of oxide‐based memristors, have been predominantly grown using traditional fabrication lines such as atomic layer deposition (ALD) and radio frequency (RF) magnetron sputtering.^[^ ^25^, ^26^, ^27^ ^]^ Recently, the easily oxidized 2D semiconductors such as SnSe_2_, MoS_2_, and black phosphorus (BP) have been intensively explored in resistive switching devices by producing a thin oxide film on the surface using ozone plasma etching method or solution process.^[^ ^28^, ^29^, ^30^ ^]^ However, the atomic layer thickness of the device can lead to tunneling phenomena, which are more complex and can occur at different bias voltages.^[^ ^31^ ^]^ This may lead to a higher off‐state current and an unstable threshold voltage, and the accumulation of metal atoms is one of the contributing factors to the reduced durability of this thinner 2D memristor.^[^ ^32^ ^]^ Hence, the integration of a single surface‐oxidized 2D memristor into logic and storage circuits still faces challenges due to the significant current in high resistance state (HRS), short retention period, and slow switching speed.^[^ ^33^, ^34^, ^35^ ^]^ Therefore, there is an urgent need for improved fabrication strategies and higher‐performing oxide memristors to optimize the manufacturing process, reduce power consumption, and enhance compatibility with the conventional semiconductor processing line.

In this work, we developed a thermal oxidation technique for controlled production of the thin TiO_x_ and SnO_x_ film from pure metallic titanium (Ti) and 2D SnSe_2_ surface, respectively, through a mild temperature annealing process. The memristors based on Ag/TiO_x_/SnO_x_/SnSe_2_ film demonstrate great potential for high‐performance read/write operations in low‐power memory systems. The device exhibits exceptional threshold resistive switching (RS) characteristics with high on/off ratio (10^6^), prolonged data retention exceeding 10^4^ s, ultra‐small subthreshold swing of 2.5 mV decade^−1^, and extremely low static power as low as 0.93 pW, that much outperforms the state‐of‐the‐art memristors relying on oxygen vacancy conductive filaments.^[^ ^34^, ^35^ ^]^ Furthermore, the thermal oxide memristor is integrated in conjunction with 2H‐MoTe_2_ transistor to establish a 1T1R structure, achieving multi‐value storage and selector function.^[^ ^33^, ^34^, ^36^, ^37^, ^38^, ^39^, ^40^ ^]^ This encompasses gate voltage modulation of the LRS resistance value and reduces the current required to maintain the LRS state, which can enable low power consumption, high‐density integration, and five distinct storage states. The memristor can be selected by the transistor as the memory unit through gate modulation to implement the selector function. Meanwhile, the formation of the p‐n junction between n‐type SnSe_2_ and p‐type 2H‐MoTe_2_ endows the device with unidirectional conduction capabilities, safeguarding the memristor against potential damage caused by negative voltage spikes and suppressing the possibly occurring sneak path current in the resistive random access memory (RRAM) crossbar array.^[^ ^41^, ^42^ ^]^ This work provides a low‐cost, scalable, and CMOS‐compatible process for high‐performance and high‐stability memristors, paving the way for high‐density data storage and in‐memory computing application.

## 2 Results and Discussion

### 2.1 Thermal Oxidation Process and Memristor Performance

**Figure 1:**
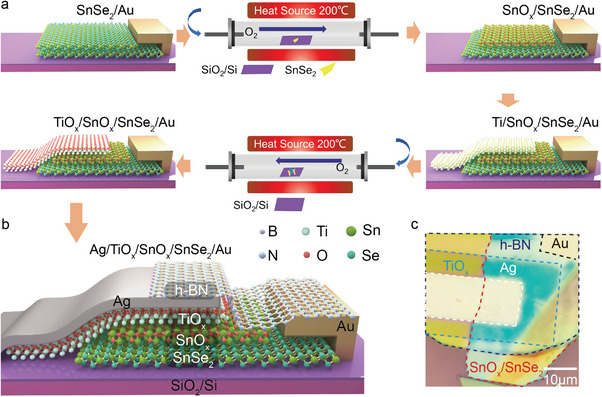
Ag/TiO_x_/SnO_x_/SnSe_2_ memristor and manufacturing process. a) Step‐by‐step process of the device fabrication. b) Schematic diagram of the memristor structure. c) Optical image of the device.

The fabrication process of the Ag/TiO_x_/SnO_x_/SnSe_2_ memristor is shown in **Figure** **1**a (see details in the Experimental Section). Briefly, the fabricated multilayer SnSe_2_‐based device was oxidized in a tube furnace at 200 °C for 1 h under a highly concentrated oxygen environment, resulting in the formation of a thin SnO_x_ layer on the surface of the SnSe_2_ channel (Figure S1, Supporting Information shows the morphology and performance difference with varying oxidation times). Subsequently, a 10 nm thick Ti layer was deposited onto the SnO_x_ surface using an electron beam evaporation technique. Then, the device was again exposed to oxidation in a tube furnace at 200 °C for 1 h, yielding the formation of TiO_x_ layer. Thereafter, a complete memristor was achieved by depositing 50 nm Ag electrodes onto the top TiO_x_ surface. Finally, the memristor was encapsulated by h‐BN to prevent damage from water molecules and atmospheric oxygen in air (Figure S2, Supporting Information shows the damage of the device without h‐BN encapsulation). Figure 1b,c show the schematic diagram and optical microscope image of the memristor based on Ag/TiO_x_/SnO_x_/SnSe_2_/Au architecture, respectively. In this architecture, the amorphous oxides (TiO_x_ and SnO_x_) with abundant oxygen vacancies play a crucial role in the resistive switching properties of the device. These oxide films are grown by thermally oxidized pure Ti and 2D SnSe_2_ at mild temperature, which can lead to a self‐assembled oxidation layer and atomic‐smooth interface, favorable for the formation/rupture of oxygen vacancy conductive filaments, yielding high threshold switching performance. Additionally, compared to the traditional oxide‐grown techniques such as ALD or sputtering, our thermal oxidation process shows the advantages of low temperature, low cost, nondestructive, CMOS compatibility, and high device yield.

**Figure 2:**
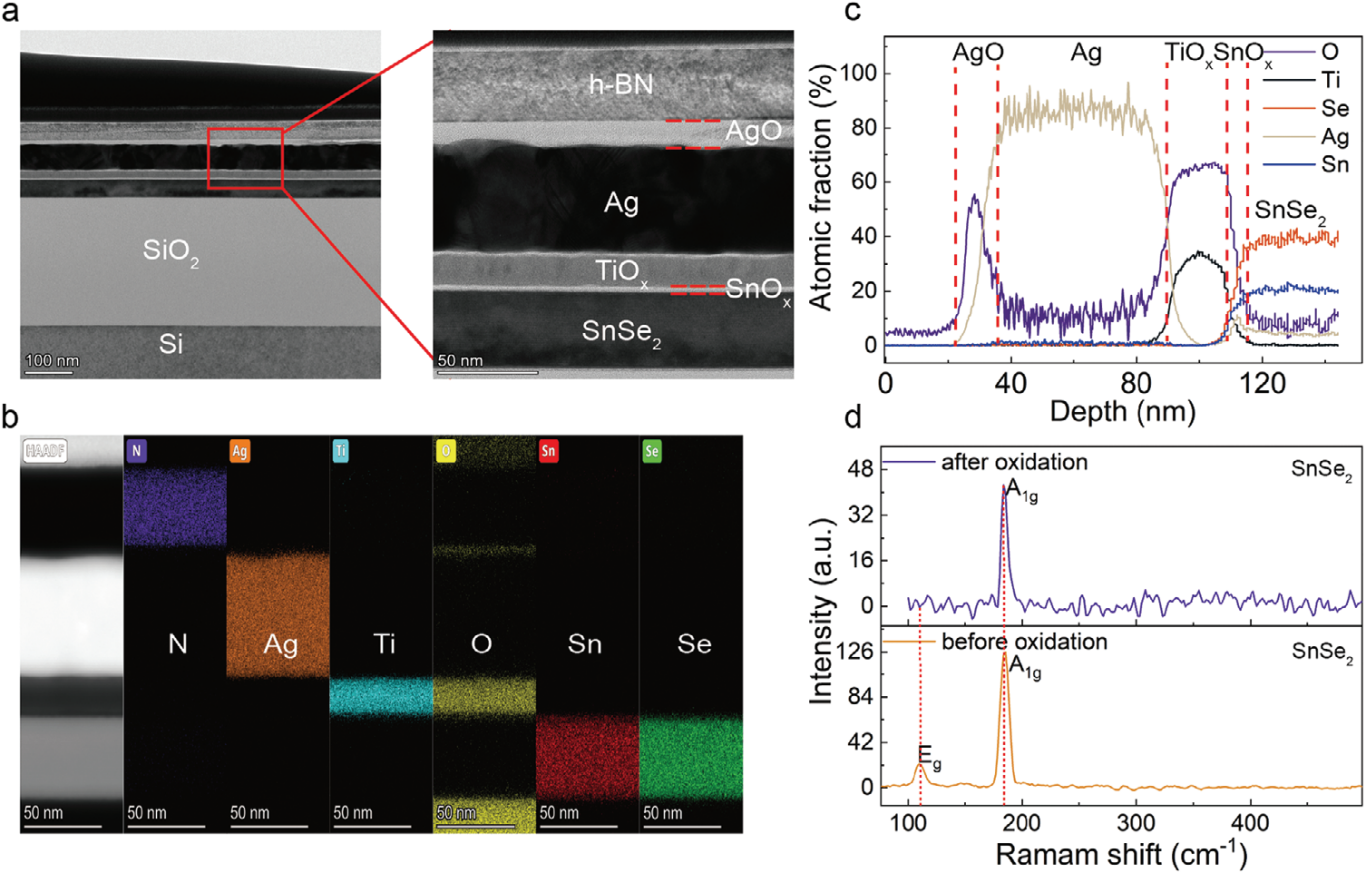
Characterizations of Ag/TiO_x_/SnO_x_/SnSe_2_ memristor. a) Cross‐sectional TEM image shows memristor architecture. b) EDS line scans of the cross‐section location. c) Cross‐sectional TEM elemental distribution. d) Raman spectra of SnSe_2_ pre‐ and post‐oxidation.

To visualize the stacking components of the memristor, the cross‐sectional profile is extracted by focused ion beam‐assisted transmission electron microscopy (FIB‐TEM). As shown in **Figure** **2**a, the different components of h‐BN (35 nm), Ag (52 nm), TiO_x_ (18 nm), SnO_x_ (4 nm), and SnSe_2_ (38 nm) can be clearly distinguished, showing the atomic‐level smooth interface between each layer. The energy‐dispersive X‐ray spectroscopy (EDX) elemental mapping is shown in Figure 2b, corresponding to the h‐BN/Ag/TiO_x_/SnO_x_/SnSe_2_ assembling from top to bottom in the device. Figure 2c shows a percentage variation of different elements along the vertical distribution, further illustrating the stacking components and the presence of a thin oxide layer of SnO_x_ on the surface of SnSe_2_. The Raman spectrum of SnSe_2_ before and after oxidation is also measured as shown in Figure 2d, the disappearance of the E_g_ mode (110 cm^−1^) and the weakening A_1g_ (185 cm^−1^) peak can be observed, indicating the oxidation of SnSe_2_ surface by the mild‐temperature thermal oxidation process.^[^ ^43^ ^]^

**Figure 3:**
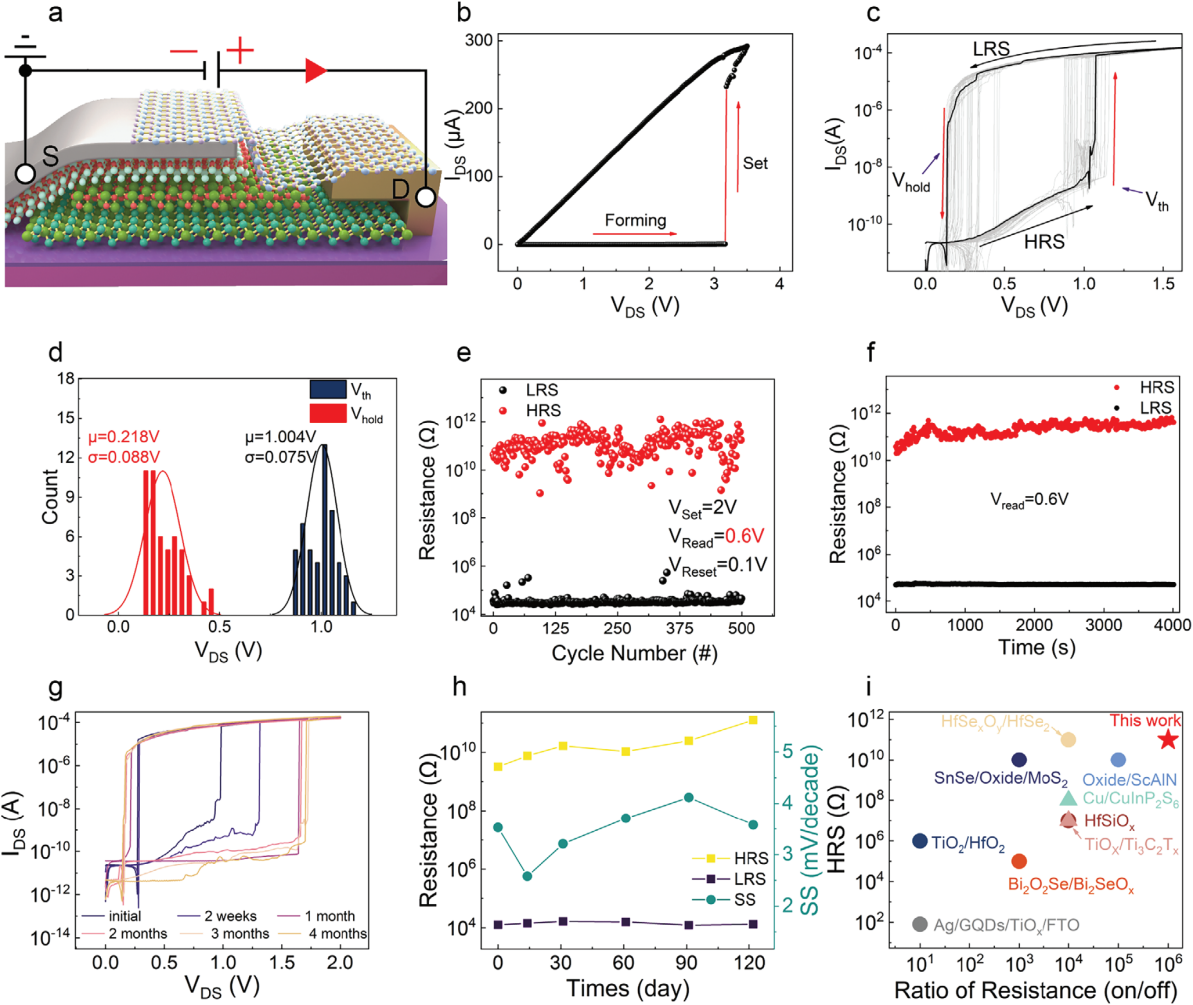
The electrical characteristic of the Ag/TiO_x_/SnO_x_/SnSe_2_ memristor. a) Schematic diagram of circuit connection for memristor measurement. b) Forming process. The inset is a diagram labeling the source‐drain location of the device structure. c) *I–V* curves for 50 consecutive cycles in DC logarithmic modes. d) Histogram of Set and Reset voltages for 50 cycles. The threshold and hold voltages have averages (μ) of 1.004 and 0.218 V with standard deviations (σ) of 0.075 and 0.088 V, respectively. e) Endurance test of 500 cycles at a read voltage of 0.6 V. f) Retention test of LRS and HRS over 4000 s. g) Stability test of the device over 4 months. h) Variation in subthreshold swing (SS) and resistance of memristor (HRS and LRS) at 0.6 V read voltage over 4 months. i) Comparison of the switching ratios and HRS of different memristors (circles represent vacancy memristors, triangles represent active cation memristors).

The electrical measurements of the Ag/TiO_x_/SnO_x_/SnSe_2_/Au memristors have been performed as shown in **Figure** **3**. In order to initiate switching behavior, the localized conductive filaments must first be formed in this device, and the measurement circuit is linked as shown in Figure 3a. Thus, we initially sweep a drain voltage from 0 to 3.5 V, the current remains off at a small bias range and increases sharply at 3.15 V due to the formation of the conductive filament as shown in Figure 3b. As a result, the device transitions from a high resistance state (HRS) to a low resistance state (LRS). At the same time, it is evident that the current of LRS during the forming process does not exceed 1 mA, implying that SnSe_2_ could be utilized as a pull‐up resistor for safeguarding the Ag/TiO_x_/SnO_x_ memristor layer. Therefore, this device does not need the function of current compliance by a semiconductor parameter analyzer.^[^ ^44^, ^45^ ^]^ After the formation process, the memristor shows typical unipolar threshold resistive switching behavior with a large hysteresis window for 50 cycles as shown in Figure 3c. The threshold and hold voltages for 50 cycles are extracted and fitted using the Gaussian equation as shown in Figure 3d, a very low average *V*_th_ of 1.004 V and *V*_hold_ of 0.2183 V can be obtained, in favor of low‐power electronic applications. The cyclic endurance under pulse‐voltage operation is also examined in threshold switching mode, demonstrating a large ON/OFF ratio of ≈10^6^ as shown in Figure 3e. The stable low‐resistance state (LRS) indicates that the integrated SnSe_2_ layer not only serves as a source for the SnO_x_ oxide layer but also functions as a protective pull‐up resistor, which provides robust protection to the resistive switching layer within the memristor and limits the emergence of a strong current. As shown in Figure 3f, both the LRS and HRS remain constant over a time of 4000 s, indicating its reliable retention and storage feature (The retention measurement method for LRS is shown in Figure S3, Supporting Information). As control experiments, the individual TiO_x_ and TiO_x_/SnSe_2_ devices are also fabricated and measured as shown in Figures S4 and S5 (Supporting Information), respectively, further implying the superiority of memristors based on thermally oxide Ag/TiO_x_/SnO_x_/SnSe_2_ structure in terms of the memory window stability and ON /OFF ratio. Meanwhile, we also fabricated the completely vertical structure of Ag/TiO_x_/SnO_x_/SnSe_2_/Graphene, as shown in (Figure S6, Supporting Information). The *I–V* measurement of the vertical structure reveals that the resistive switching primarily hinges upon the Ag/TiO_x_/SnO_x_ layer.

To evaluate the long‐term air‐stability of the memristor with h‐BN encapsulation, a series of resistive switching (RS) measurements are performed at various time intervals spanning four months as depicted in Figure 3g. The *V*_Set_ value gradually increases over time and eventually stabilizes at 1.6 V after 4 months, which can be attributed to a dynamic equilibrium of oxygen vacancies between TiO_x_ and SnO_x_ as time progresses. Figure 3h shows the subthreshold swing (SS) and resistance values of HRS/LRS (at a reading voltage of 0.6 V) as a function of times up to 4 months, the SS and ON/OFF ratios remain less than 4 mV decade^−1^ (minimum value of 2.5 mV decade^−1^) and ≈10^6^, respectively, suggesting an excellent switching behavior with ultra‐steep slope, high ON/OFF ratio, and high air‐stability. The performance comparison is shown in Figure 3i, our device exhibits the highest HRS and ON/OFF ratio among the latest developed vacancy‐ and cation‐based memristors, endowing our device with more promising potential for low‐power memory applications.^[^ ^22^, ^35^, ^46^, ^47^, ^48^, ^49^, ^50^, ^51^, ^52^ ^]^ Meanwhile, the SS performance of the memristor is compared with the current memristor as shown (Figure S7, Supporting Information), indicating that our device has an ultra‐steep slope. In addition, more than ten devices fabricated using the same thermal oxidation process have demonstrated highly reproducible threshold switching performance (Figure S8, Supporting Information), indicating the good device‐to‐device stability and high device yield by this method.

### 2.2 Mechanism of Threshold RS Behavior

**Figure 4:**
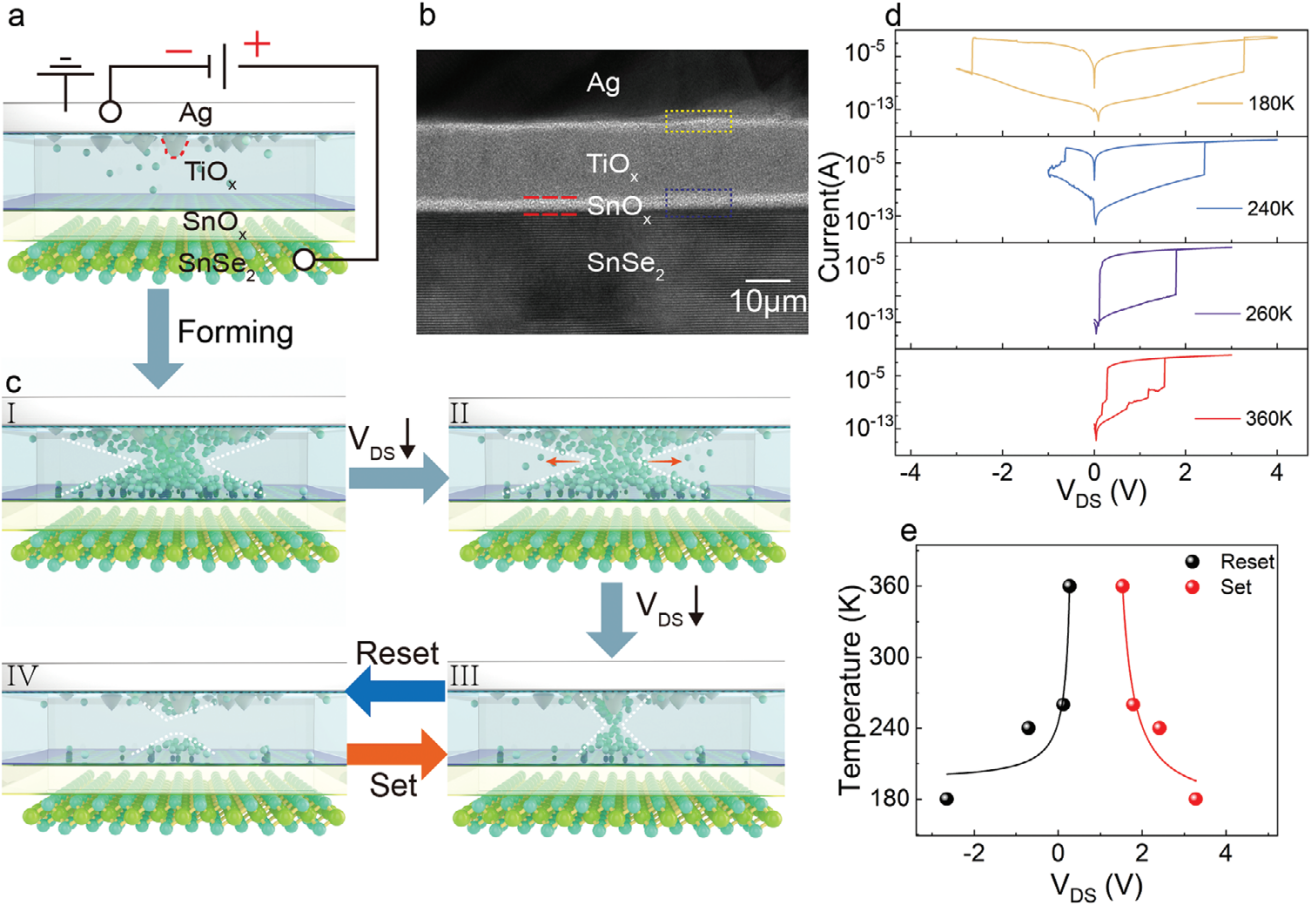
The resistive switching mechanism of the Ag/TiO_x_/SnO_x_/SnSe_2_ memristor. a) Schematic diagram of the memristor before forming process. b) The cross‐sectional TEM image shows the corresponding memristor architecture. c) Schematic diagram of the switching mechanism for the Set and Reset processes. d) *I–V* curves of Ag/TiO_x_/SnO_x_/SnSe_2_/Au memristor measured from 180 to 360 K. e) Temperature‐dependent Set/Reset voltages in the memristor.

The mechanism of the oxide‐based memristor is generally deduced to the migration and diffusion of oxygen vacancies.^[^ ^23^, ^53^ ^]^ Also, the *I–V* measurements of various metal contact electrodes reveal that Ag plays a crucial role in the performance of memristors, as shown in Figure S9 (Supporting Information). To investigate the RS mechanism of our thermally oxidized memristor, the cross‐sectional 3D diagram and the circuit are depicted in **Figure** **4**a. Meanwhile, we discerned that the TiO_x_ layer incorporates Ag element in the cross‐sectional TEM elemental distribution, as shown in Figure 2c (at 90 nm). Therefore, in the schematic depicted in Figure 4a, the Ag clusters have been incorporated at the Ag‐TiO_x_ interface to facilitate a more comprehensive understanding of the resistance‐switching mechanism of our device. From the FIB‐TEM image (Figure 4b), the oxide layer consists of TiO_x_ and SnO_x_ thin films where abundant oxygen vacancies exist due to their amorphous phase. During the forming process, a scanning voltage ranging from 0 to 3.5 V is applied to the memristor, which can induce the migration of oxygen vacancies in SnO_x_ layer toward TiO_x_ and generate additional oxygen (O) vacancies at Ag‐TiO_x_ interface (as shown in the yellow dashed box of Figure 4b) by drawing O atoms from TiO_x_.^[^ ^35^ ^]^ This will cause the oxygen vacancies conductive filament near the Ag‐TiO_x_ interface to form a stable capillary bridge by the minimization of the surface energy of Ag clusters.^[^ ^54^, ^55^ ^]^ Then the oxygen vacancy defects in TiO_x_ layer and the Ag+ cations collaborate to form charge‐transfer channels, which facilitate the migration of oxygen vacancies and serve as electronic hopping points when an electrical voltage is applied.^[^ ^56^ ^]^ And eventually, combine with the oxygen vacancies migrated from SnO_x_ to form a conductive channel between the Ag electrode and SnSe_2_, as illustrated in Figure 4c‐I. Consequently, the device resistance transitions from a high‐resistance state (HRS) to a low‐resistance state (LRS). At lower voltages, the electric field intensity is insufficient to sustain the conductive filament state due to the significantly higher concentration of oxygen vacancies within the conductive filament than its surrounding environment, resulting in diffusion of oxygen vacancies (as illustrated in the red arrow of Figure 4c‐II). The generated Joule heating at the LRS can also induce the fracture of the conductive filament at their thinnest regions, thereby eventually giving rise to the switchover of the device from the LRS back to the HRS (as illustrated in Figure 4c‐III,IV for Reset process).^[^ ^9^, ^57^ ^]^ As the electric field gradually increases again, the disrupted oxygen vacancies in the conductive filaments migrate and reassemble, thus forming new conductive filaments to set the memristor (as depicted in Figure 4c‐III,IV for Set process). Furthermore, to substantiate that the resistive switching behavior of the memristor is predominantly governed by oxygen‐vacancy conductive filaments and the synergistic effect of Ag conductive filaments at the TiO_x_ interface, a negative voltage was applied in the memristor, as depicted in (Figure S10, Supporting Information). The results suggest that the forward voltage can no longer form a threshold behavior after applying a voltage of −4 V, indicating that the penetration of too many Ag ions into the resistive layer will destroy the formation and disruption of the oxygen‐vacancy conductive filaments.^[^ ^56^ ^]^

To further explore the physical model of oxygen vacancy conductive filaments, the RS *I–V* curves at different temperatures are measured and shown in Figure 4d. The variation of Reset and Set voltages with temperature is illustrated in Figure 4e. With decreasing temperature, the Set voltages (*V*_Set_) required to form a conductive filament increase. Conversely, the Reset voltages (*V*_Reset_) needed to disrupt the conductive filament shift to be negative as temperature decreases. The filament formation is theoretically governed by microscopic ionic hopping and ionization processes, characterized by rates expressed as:

(1) $$r\;=r_{0}\;\exp\left(-\frac{E_{b}-d\varepsilon }{k_{B}T}\right)$$

where 𝑟_0_ represents the rate constant, the barrier height (𝐸_b_), effective hopping distance (𝑑), and electric field along the hopping direction (𝜖) play crucial roles in this phenomenon, which has been demonstrated both analytically and experimentally.^[^ ^43^, ^58^, ^59^ ^]^ In our case, the presence of residual conductive filaments within TiO_x_ is responsible for the reduction in the potential barrier (𝐸_b_) due to the Fermi pinning effect (See the detail in Note S1, Supporting Information). This implies that a lower electric field (𝜖) is required to recover the conductive filaments compared to their initial establishment. With an increase in temperature, there is a corresponding rise in the value of r, leading to an enhancement in diffusion and migration ability, that can facilitate the formation or disruption of conductive filaments with an electric field, as depicted in Figure 4e. As a result, the device undergoes a transition from unipolar volatile to bipolar nonvolatile behavior by decreasing temperature to less than 240 K. Thus, our device exhibits nonvolatile behavior at low temperatures and unipolar threshold switching behavior at room temperature, which is attributed to the temperature‐dependent migration and diffusion of oxygen vacancies.^[^ ^50^ ^]^ This transition can endow our memristor with function flexibility depending on the atmosphere temperature and application scenario.

### 2.3 1T1R Design and Performance Measurement

**Figure 5:**
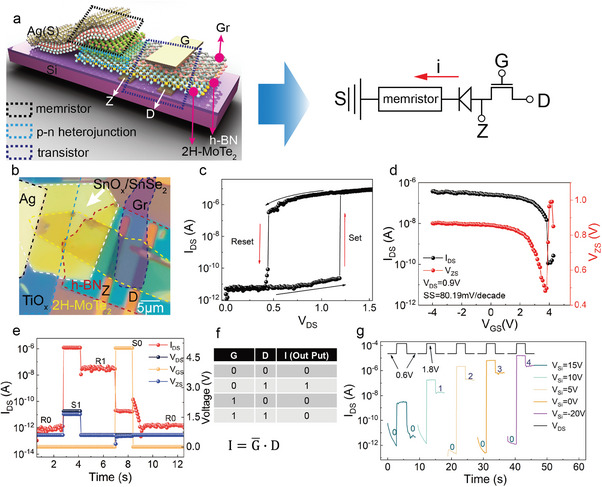
Structural and electrical characteristics of the 1T1R device. a) Schematic diagram of the 1T1R structure. b) Optical image of the device. c) Typical *I−V* switching curves of the 1T1R device. The arrows indicate the direction of hysteresis. d) Transfer curves and *V*_ZS_‐*V*_GS_ variation curves (with 80.19 mV/decade^−1^) in the 1T1R devices. e) 1T1R device performs write, erase, and read processes (S for write or erase, R for read). f) The truth table for 1T1R under the influence of *V*_GS_ and *V*_DS_, where output 1 is obtained from R1 state, and output 0 is obtained from R0 state. g) Multilevel memory operation process by utilizing back gate (*V*_Si_) pulses ranging from −20 to 15 V, with Set voltage of 1.8 V and Read voltage of 0.6 V.

The 1T1R (one transistor in conjunction with one memristor) is a particularly important electric unit to integrate the multiple functional modules into neuromorphic computing and high‐density storage systems, such as sensing‐computing integrated technology, and artificial neural networks. However, some reported 1T1R architectures usually connect a transistor and a memristor through metal electrodes, which will require an extra step in processes such as photolithography and gold plating, meaning that the complexity of the integration is increased and the process can introduce defects that can lead to degradation of the device performance.^[^ ^34^, ^60^, ^61^ ^]^ To further take advantage of our thermally oxidized memristor, we implemented a 1T1R structure by integrating the memristor with 2H‐MoTe_2_ transistor by a vdW stacking approach. The schematic diagram and optical microscopy image of the 1T1R configuration are shown in **Figure** **5**a,b, respectively. The simplified circuit diagram is depicted on the right‐hand side of Figure 5a. The source (S), drain (D), and gate (G) electrodes are assigned to the TiO_x_, 2H‐MoTe_2,_ and graphene, respectively. The additional Z electrode is located in the middle of 2H‐MoTe_2_ channel to monitor the voltage applied on the memristor. By sweeping the gate voltage (*V*_G_) from 0 to 3.5 V, the current between Z and D electrodes (*I*_DZ_) decreases and approaches zero, indicating a typical p‐type transport behavior of 2H‐MoTe_2_ transistor as shown in Figure S11 (Supporting Information). From the *I*_DS_–*V*_DS_ characteristic as shown in Figure 5c, the 1T1R device also exhibits a good threshold resistive switching performance with a threshold voltage of 1.18 V and hold voltage of 0.45 V. Figure 5d shows the voltage (*V*_ZS_) and current (*I*_DS_) in relation to the top‐gate *V*_GS_. It is observed that both *V*_ZS_ and *I*_DS_ decrease gradually by sweeping *V*_GS_ from −4 to 3.8 V, which is attributed to the increased resistance and divided voltage in 2H‐MoTe_2_ transistor. When the *V*_GS_ reaches 3.8 V, the *V*_ZS_ decreases to 0.48 V, which can reset the memristor by disrupting the oxygen‐conductive filament. Thus, the HRS of the memristor can subsequently result in an instant increase in *V*_ZS_ and simultaneously a sharp decrease in *I*_DS_ of the 1T1R device.

To illustrate the fundamental read and write functions of the 1T1R device, a write voltage of 1.8 V and an erase voltage of 0 V were applied to the D electrode to set the “1” and “0” state, respectively, as shown in Figure 5e. The logic switching ratio of this 1T1R device is demonstrated to be 10^5^. By applying a top gate voltage (*V*_TG_) of 5 V, the device can also be reset to “0” state. Based on the read/write operation results, we can derive the truth table and logic expression as shown in Figure 5f. Consequently, the memristor can be capable of storing data when the G electrode is set to a low voltage (0 V), while it always remains off state when G is set to a high voltage (5 V), allowing the G electrode to serve as an alternative selector switch similar to the role of the CS (Chip select) pin in the integrated circuit. The selector function of the 1T1R device is compatible with conventional CMOS devices,^[^ ^36^, ^37^ ^]^ and can be implemented in data erasing process of memories using a gate voltage rather than an input drain voltage, allowing a higher priority control on the state of the memristor.

Furthermore, the carrier concentration in 2H‐MoTe_2_ can be effectively regulated through back‐gate modulation, enabling additional manipulation of writing and erasure processes under distinct back‐gate voltage (*V*_Si_) at room temperature, as illustrated in Figure 5g. By applying read, write, and erase voltages of 0.6, 1.8, and 0 V at the D electrode, respectively, 1T1R device is switched on step‐by‐step with the gradual decrease of *V*_Si_: 15, 10, 5, 0, and −20 V, which correspondingly enables the device to reach the states 0, 1, 2, 3 and 4, demonstrating an effective multilevel storage capability. Compared to the reported 1T1R devices, the wider interval range of *V*_Si_ voltage for achieving multi‐value storage states is more effective in suppressing noise disturbances during circuit operation.^[^ ^34^ ^]^ If the 1T1R device stores only one byte of data, the LRS resistance can be increased to reduce power consumption by applying a *V*_Si_ of 10 V during data storage. Conversely, when reading data, the LRS resistance can be decreased by applying a *V*_Si_ voltage of −20 V to enhance the switching ratio and improve the resolution of read data. Therefore, the 1T1R device exhibits a static power of 0.93 pW and 20.82 nW at data 0 and 1, respectively, according to the following equations:

(2) $$P_{\mathrm{Static}}=V_{\mathrm{DS}}\;\times I_{\mathrm{DS}}$$

where *V*_DS_ represents the read voltage, *I*_DS_ is the current reading value. To be in contrast, the static power of the memristor without 2H‐MoTe_2_ is measured to be 28.4 µW at data 1, more than three orders of magnitude higher than that in 1T1R device. This further demonstrates the gate erasability and ultra‐low power consumption capabilities of this memristor in conjunction with 2H‐MoTe_2_ transistor. Table S1 (Supporting Information) shows the comparison of performance against the reported 2D memtransistor, demonstrating the higher performance and lower power consumption of our device. Additionally, the 2H‐MoTe_2_/SnSe_2_ p‐n heterojunction in 1T1R device exhibits a current rectification behavior and unidirectional conductivity (Figure S11, Supporting Information), effectively safeguarding against potential damage caused by large reverse voltages to the memristor.

## 3 Conclusion

In summary, this work proposes a low‐cost and CMOS‐compatible thermal oxidation process for the fabrication of low‐power and ultra‐steep memristors with SS of 2.5 mV decade^−1^. The TiO_x_ and SnO_x_ thin films with abundant oxygen vacancies can be grown by thermally oxidized pure Ti and 2D SnSe_2_ at mild temperatures. Due to the self‐assembled oxidation layer and atomic‐smooth interface, the memristor exhibits exceptional threshold switching performance with a high ON/OFF ratio of 10^6^, low threshold/hold voltage, and stable cyclic endurance and retention. A physical model of oxygen vacancy conductive filaments is proposed through the FIB‐TEM and variable‐temperature *I–V* characterization to explain the resistive switching behavior of the device. A transition between unipolar volatile and bipolar nonvolatile characteristics is also observed in this memristor by varying temperatures. The 1T1R device is further designed by integrating the thermally oxidized memristor with 2H‐MoTe_2_ transistor, expanding its potential applications for selector functions and multi‐value storage with ultra‐low power consumption. This work develops a facile and useful thermal oxidation technique for high‐performance and high‐stability oxide‐based memristors and offers a novel concept of 1T1R design with a vdW stacking method for low‐power high‐density data storage and in‐memory computing applications.

## 4 Experimental Section

### Device Fabrication

The multilayer SnSe_2_ was obtained through mechanical exfoliation from the bulk crystal (Shanghai OnWay Technology Co., Ltd). Subsequently, the SnSe_2_ flakes were transferred onto the Si substrate with a 300 nm SiO_2_ layer. The SiO_2_/Si substrate with samples was spin‐coated with a photoresist (An ARP‐5350 positive photoresist was supplied by Taizhou SUNANO New Energy Co., Ltd) at 4000 rpm for 60 s and baked at 100 °C for 4 min. A 50 nm Au electrode was deposited on one side of SnSe_2_ using the ultraviolet maskless photolithography machine (TuoTuo Technology Co., Ltd) and electron beam vaporization. The SnSe_2_ device was then oxidized in a tube furnace at 200 °C for 1 h under a highly concentrated oxygen environment, forming a thin SnO_x_ film on the surface of SnSe_2_. A 10 nm thick Ti layer was deposited onto the SnO_x_ surface using an electron beam evaporation technique, which was then oxidized to be TiO_x_ layer at 200 °C for 1 h in a tube furnace. A 50 nm Ag electrode was deposited on top of TiO_x_ layer. As such, the memristor based on Ag/TiO_x_/SnO_x_/SnSe_2_/Au architecture was achieved. Finally, the device was encapsulated by h‐BN.

### Characterization

The device was analyzed using STEM and energy‐dispersive X‐ray spectroscopy (EDX) elemental mapping on a ThermoFisher talos F200X instrument. The Raman spectra were acquired using a Thermo DXR2xi Raman spectrometer system (LabRAM HR Evolution, HORIBA Jobin Yvon), excited by a continuous wavelength 532 nm laser with a spot diameter of 1–2 µm. The devices were electrically characterized using a probe station (PSAICPB6A, Precision Systems Industrial Co., Ltd.) equipped with a Keithley 2636B and 2611B semiconductor source meter. The variable temperature *I–V* properties of films were characterized using a high‐precision semiconductor analyzer (Agilent B1500A).

## Conflict of Interest

The authors declare no conflict of interest.

## Supporting information

Supporting Information

## Data Availability

The data that support the findings of this study are available from the corresponding author upon reasonable request.
