# Supplementary material for: Thermally Oxidized Memristor and 1T1R Integration for Selector Function and Low‐Power Memory
Source: Adv Sci (Weinh). 2024 Jul 3;11(33):2401915. doi: 10.1002/advs.202401915 (PMC11434030; doi:10.1002/advs.202401915)
Supplement: Supplementary file 1 — Supporting Information [file ADVS-11-2401915-s001.docx]

Supporting information

**Thermally oxidized** **memristor and 1T1R integration for selector function and low-power memory**

*Zhidong Pan^1^, Jielian Zhang^1^, Xueting Liu^1^, Lei Zhao^1^, Jingyi Ma^1^, Chunlai Luo^2^, Yiming Sun^1^, Zhiying Dan^1^, Wei Gao^1^, Xubing Lu^2^, Jingbo Li^3^, Nengjie Huo^1,4^**

^1^School of Semiconductor Science and Technology, South China Normal University, Foshan 528225, P.R. China

^2^School of South China Academy of Advanced Optoelectronics, South China Normal University, Guangzhou 510006, P.R. China

^3^College of Optical Science and Engineering, Zhejiang University, Hangzhou 310027, P.R. China

^4^Guangdong Provincial Key Laboratory of Chip and Integration Technology, Guangzhou 510631, P.R. China.

*Corresponding author

E-mail: njhuo@m.scnu.edu.cn

**Table S1.** Comparison of performance and phenomena with previously reported memtransistors articles

| Different devices | Threshold voltage | On/Off ratio | Current of HRS | application | Ref |
| --- | --- | --- | --- | --- | --- |
| MoS_2_ | Very high | 10^3^ | 0.1nA | neuromorphic learning | ^[1]^ |
| WSe_2_ | high | 10^1^ | 10nA | neural network | ^[2]^ |
| MoS_2_ | Very high | 10^3^ | 1pA | collision detector | ^[3]^ |
| Our device | low | 10^5^ | 1pA | Low power memory | This work |

**Note S1:**

After the forming process, the defect energy levels will create by the remaining oxygen vacancy conducting filaments, in conjunction with the impurity energy levels of the doped Ag, which were penetrated into the Ag-TiO_x_ interface by the thermal evaporation, expand the surface state density region. This leads to a reduction in the metal-semiconductor contact barrier and results in filling of the defect/impurity energy level with electrons or holes, thereby generating a Fermi energy level pinning effect.^[4]^ This will facilitate electron passage through the Schottky barriers at lower electric fields, making oxygen vacancy ionization leaps and oxygen vacancy conducting filaments easier to be formed at lower Set voltages, as can be concluded from the I-V measurement results before and after Forming process.


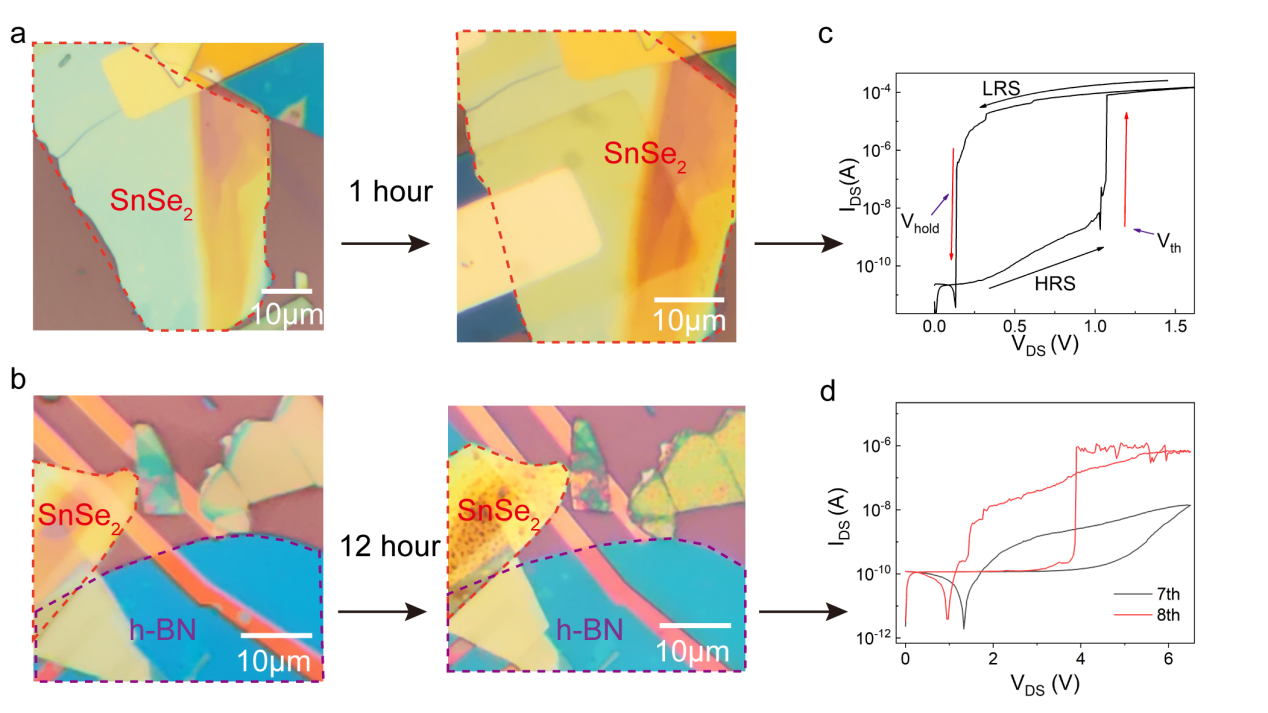


**Figure S1. Optical microscopy images and I-V measurement results of the corresponding memristors with different oxidation times.** (a) Devices oxidised for one hour. (b) Devices oxidised for 12 hours. (c) I-V measurement results of memristor for one hour oxidation. (d) I-V measurement results of memristor for 12 hours oxidation.


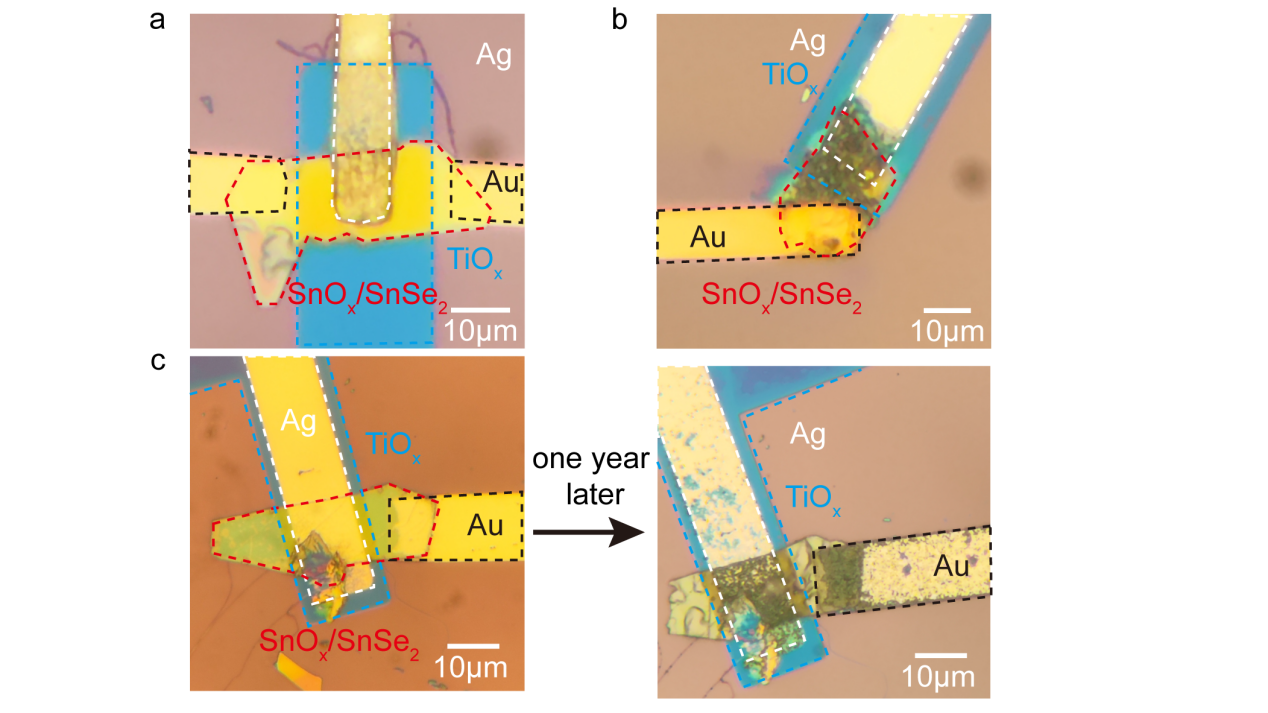


**Figure S2. Memristor without h-BN encapsulation.** (a) Placed for two to three weeks. (b) Placed for more than six months. (c) Difference between devices after one month and one year.





**Figure S3**. LRS long-term retention performance Test (The inset illustrates the first 13 seconds of the test, showcasing the device's initial state as HRS, transitioning to LRS following a 2V write voltage).

**The method of LRS long-term retention measurement:** Firstly, setting the read voltage to 0.6V to capture the initial state of the memristor, then switching it from HRS to LRS by setting a write voltage of 2V, and finally applying a voltage of 0.6V to read the data to observe its maintenance ability, as shown in inset of Figure S3.


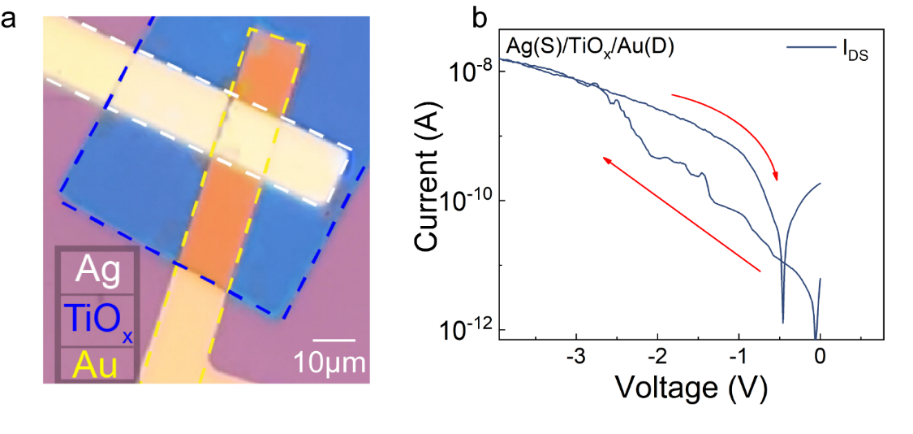


**Figure S4. Optical microscope images and I-V characteristics of the Ag(S)/TiO_x_/Au(D) device.** (a) The optical image of the device. (b) Characterization of the I-V measurement of the device.


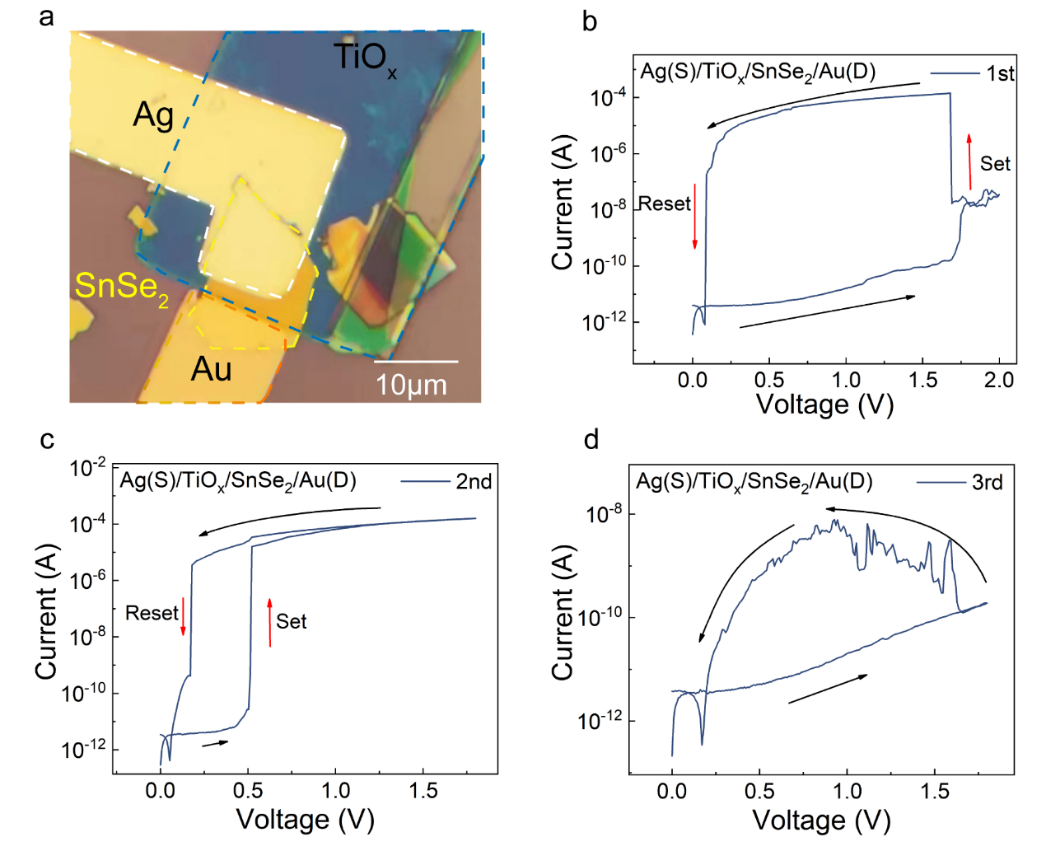


**Figure S5.** **Optical microscope images and I-V characteristics of the Ag(S)/TiO_x_/SnSe_2_/Au(D) device.** (a) Optical image of the device. (b) Characterization of the first I-V hysteresis window of the device. (c) Characterization of the second I-V hysteresis window of the device. (d) Characterization of the third I-V hysteresis window of the device.

The absence of the SnO_x_ layer in the device results in a reduced repeatability and deteriorating performance as shown in Figure S5. The insufficient provision of oxygen vacancies caused by the absence of the SnO_x_ layer is responsible for this phenomenon, leading to a gradual migration of conductive filaments from the lower part upwards under an electric field. Consequently, a disconnection occurs between the conductive filaments and the SnSe_2_ layer, resulting in an inability to form stable conductive filaments and ultimately reducing repeatability (as also evidenced by Figure S5d).


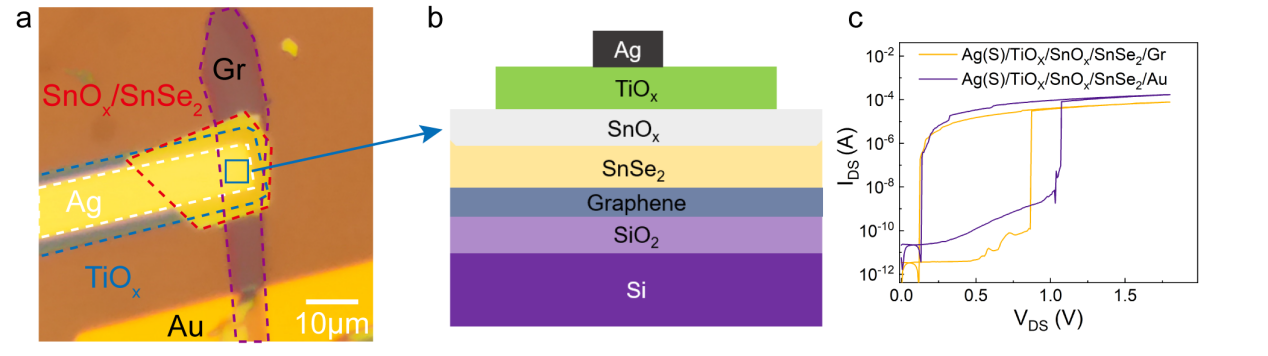


**Figure S6. Vertical structure memristor.** (a) Optical microscope images of Ag/TiO_x_/SnO_x_/SnSe_2_/Gr vertical structures. (b) Cross-section schematic diagram of the Ag/TiO_x_/SnO_x_/SnSe_2_/Gr device. (c) Comparative I-V measurement diagrams for devices corresponding to vertical and containing lateral transport.

The results of the I-V measurement are similar to the performance of the lateral structure (Ag/TiO_x_/SnO_x_/SnSe_2_/Au), as shown in Figure S6c. The results demonstrate that SnSe_2_ exhibits excellent electrical conductivity and can serve as a low resistance conductive layer material without adversely impacting the resistive layer. The reason for this can be attributed to both the degenerate n-type semiconductor property and the metallic behavior exhibited by SnSe_2_.^[5]^ Furthermore, as shown in Figure S6b and 6c, the vertically transported section of SnSe_2_ also functions as a pull-up resistor in the vertical configuration. Hence, the lateral transport of SnSe_2_ does not significantly impact the overall structure of the memristor, indicating that the resistive-switching in the memristor is primarily attributed by the Ag/TiO_x_/SnO_x_ structure.


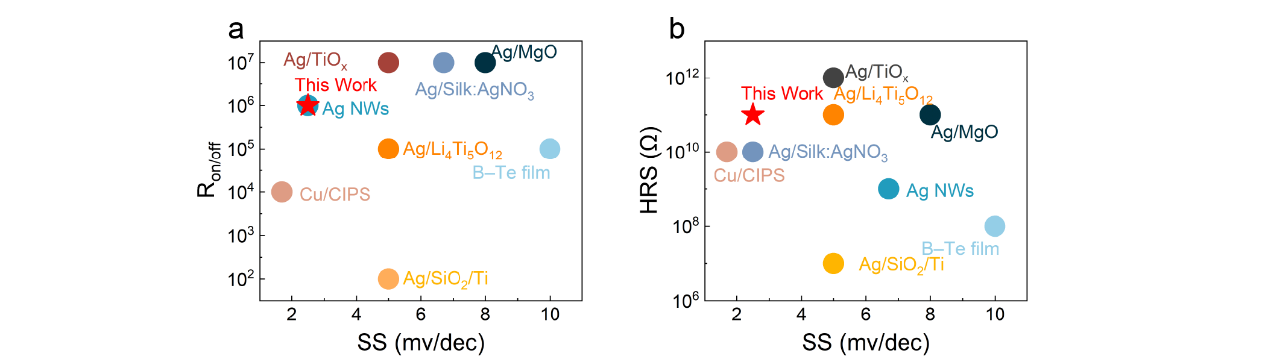


**Figure S7. the SS performance of the current memristors.** (a) Comparison of the switching ratios and SS of different memristors. (b) Comparison of the HRS and SS of different memristors.^[6-13]^


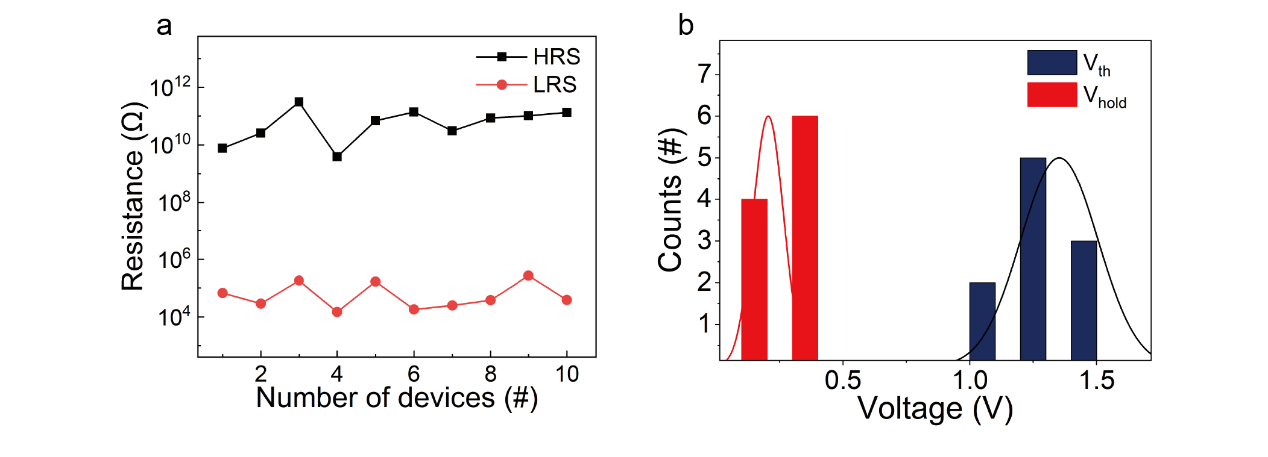


**Figure S8. Fundamental properties of different devices using the same fabrication process.** (a) The HRS and LRS data from ten devices, with a reading voltage of 0.6 V. (b) The threshold and hold voltage values from ten devices follow a Gaussian distribution.


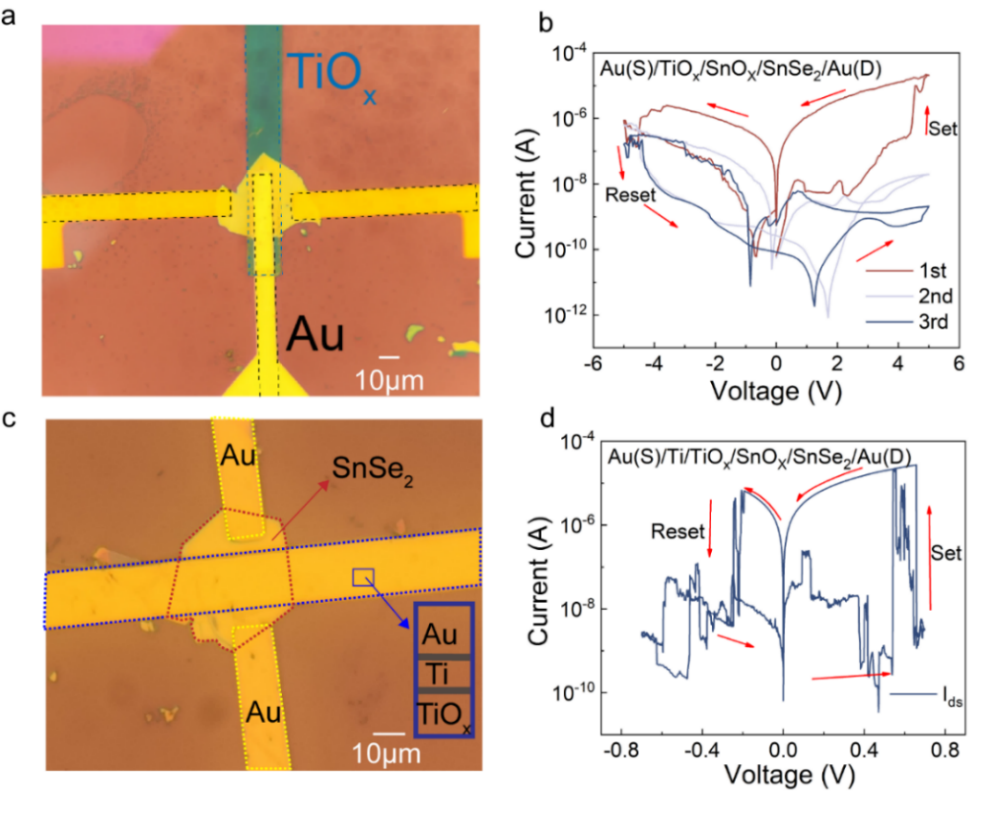


**Figure S9. Optical microscope images and I-V characteristics of different contact electrodes located on the TiO_x_ surface.** (a) Optical image of the Au(S)/TiO_x_/SnO_x_/SnSe_2_/Au(D) device. (b) I-V characteristics of the Au(s)/TiO_x_/SnO_x_/SnSe_2_/Au (three times). (c) Optical image of the Au(S)/Ti/TiO_x_/SnO_x_/SnSe_2_/Au(D) device. (d) I-V characteristics the Au(S)/Ti/TiOx/SnOx/SnSe2/Au(D) device.

The results demonstrate that the Au-TiO_x_ system requires a higher set and reset voltage and exhibit an unstable switching ratio, and the Ti-TiO_x_ system displays a lower set and reset voltage but poor stability in maintaining high resistance values, as shown in Figure S9. The comparison experiment discussed herein emphasizes the pivotal role of Ag in this memristor and simultaneously demonstrates the significant contribution of Ag in the generation and accumulation of oxygen vacancies.





**Figure S10.** The I-V measurements comprise of three sets of data before and after the application of -4V, respectively.

The device exhibits unipolar resistive switching behavior before applying -4V voltage. The resistance of the device diminishes upon application of -4V, suggesting that this voltage induces ionization of Ag, leading to the formation of Ag ions. These ions subsequently migrate into the TiO_x_/SnO_x_ layer, resulting in the creation of Ag conducting filaments. This can be explained by the instantaneous increase in current at a voltage of -3.5 V, as shown in Figure S10. Meanwhile, there is no resistive switching behavior in the forward direction after applying -4 V, indicating that the excessive Ag conductive filament will destroy the oxygen vacancy resistive switching behavior in resistive layer, which indicates that the resistive switching behavior of Ag/TiO_x_/SnO_x_/SnSe_2_ memristor is dominated by oxygen-vacancy and synergized by Ag conductive filaments at the Ag/TiO_x_ interface. In summary, the resistive switching mechanism discussed in this paper is primarily governed by oxygen vacancies, with Ag ions playing a notably significant role in Ag/TiO_x_ as analyzed in the original paper.


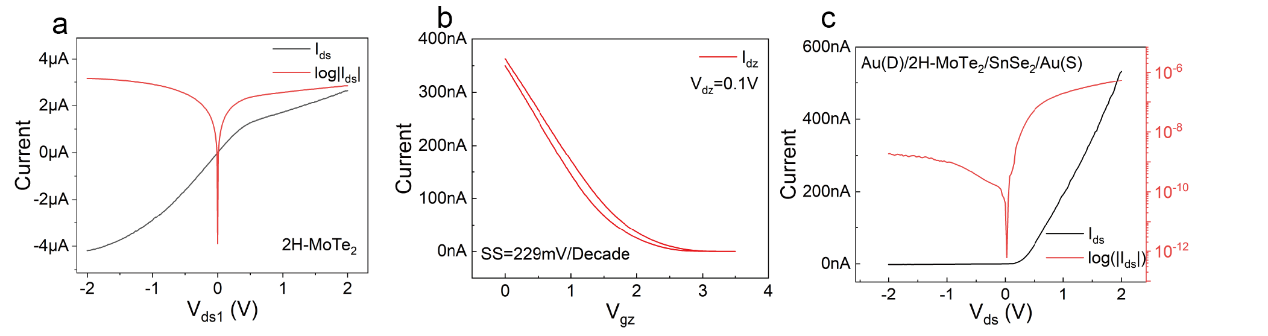


**Figure S11. Electrical characterization of 2H-MoTe_2_ and SnSe_2_.** (a) I-V characteristics of 2H-MoTe_2_ transistor. (b) Transfer curves for a 2H-MoTe_2_ transistor (The 2H-MoTe_2_ demonstrates exceptional gate modulation capabilities and 229 mv/decade). (c) I-V characteristics of 2H-MoTe_2_/SnSe_2_ junction, exhibiting a rectifying behavior.

**Reference:**

[1] V. K. Sangwan, H.-S. Lee, H. Bergeron, I. Balla, M. E. Beck, K.-S. Chen, M. C. Hersam, *Nature* **2018**, 554, 500.

[2] J. F. Leong, Z. Fang, M. Sivan, J. Pan, B. Tang, E. Zamburg, A. V. Y. Thean, *Adv. Funct. Mater.* **2023**, 33, 2302949.

[3] D. Jayachandran, A. Pannone, M. Das, T. F. Schranghamer, D. Sen, S. Das, *ACS Nano* **2022**, 17, 1068.

[4] X. Liu, M. S. Choi, E. Hwang, W. J. Yoo, J. Sun, *Adv. Mater.* **2022**, 34, 2108425.

[5] C. Guo, Z. Tian, Y. Xiao, Q. Mi, J. Xue, *Appl. Phys. Lett.* **2016**, 109, 203104.

[6] J. Yoo, J. Park, J. Song, S. Lim, H. Hwang, *Appl. Phys. Lett.* **2017**, 111.

[7] X. Huang, R. Fang, C. Yang, K. Fu, H. Fu, H. Chen, T.-H. Yang, J. Zhou, J. Montes, M. Kozicki, *Nanotechnology* **2019**, 30, 215201.

[8] Y. Sun, C. Song, S. Yin, L. Qiao, Q. Wan, R. Wang, F. Zeng, F. Pan, *Adv. Electron. Mater.* **2020**, 6, 2000695.

[9] J. Sun, H. Wang, F. Song, Z. Wang, B. Dang, M. Yang, H. Gao, X. Ma, Y. Hao, *Small* **2018**, 14, 1800945.

[10] J. Yoo, D. Lee, J. Park, J. Song, H. Hwang, *IEEE J. Electron Devices Soc.* **2018**, 6, 821.

[11] D. Wang, S. Zhao, L. Li, L. Wang, S. Cui, S. Wang, Z. Lou, G. Shen, *Adv. Funct. Mater.* **2022**, 32, 2200241.

[12] M. Zhao, S. Wang, D. Li, R. Wang, F. Li, M. Wu, K. Liang, H. Ren, X. Zheng, C. Guo, *Adv. Electron. Mater.* **2022**, 8, 2101139.

[13] Z. Zhong, S. Wu, X. Li, Z. Wang, Q. Yang, B. Huang, Y. Chen, X. Wang, T. Lin, H. Shen, X. Meng, M. Wang, W. Shi, J. Wang, J. Chu, H. Huang, *ACS Nano* **2023**, 17, 12563.
